# Epigæa: A Proving

**Published:** 1881-10

**Authors:** C. F. Millspaugh

**Affiliations:** Binghamton, N. Y.


### EPIGÆA.

C. F. Millspaugh, M. D., Binghamton, N. Y.

Epigæa repens. Linn.

Family, Ericaceæ.—The Heaths.

Tribe, *Andromedeæ.*

Common names: Ground laurel, Trailing Arbutus, May-flower, Gravel-plant.

Botanical relatives: Gaultheria, Kalmia, Rhododendron, Ledum, Uva-ursi.

Description: A rusty, hairy, creeping plant; flowering in early spring, sometimes even under the snow. The blossoms are pink, salver-form, with the tube hirsute inside, and exhale a rich, spicy odor. Leaves, rounded heart-shaped, alternate upon the stem, those upon the plant while flowering being the leaves of the previous year.

Habitat: Rich, sandy woods, preferring damp, mossy banks under evergreens.

Preparation: The roots, leaves and stems, we use for making the tincture, while the plant is budding to blossom. These parts are cut up and macerated for fourteen days in twice their weight of strong alcohol, shaking twice a day, the menstruum being then filtered off, forms a dark brown, astringent, acid tincture, of a woody odor.

Chemistry: *Tannin* in the proportion of 3.5 %, the glucosides, *urson, ericolin* and *arbutin;* a pale yellow aromatic oil, *ericinol* $C_{10}H_{16}O$, and *formic acid*, have been determined in this plant, as well as in its several relatives.

After washing the alcoholic extract of the leaves with water and ether, boiling alcohol extracts from the residue *urson* $C_{20}H_{34}O_2$; this crystallizes in tasteless, silky, fusible and sublimable needles, is insoluble in water, dilute acids and alkalies, and is sparingly soluble in ether and cold alcohol.

*Ericolin*, $C_{34}H_{56}O_{21}$, is a brown-yellow, bitter glucoside, soluble in water, alcohol, and in alcoholic ether, and not precipitable by lead salts.

*Arbutin*, $C_{12}H_{16}O_7$, is obtained in the same manner as *salicin* from willow bark; by exhausting the leaves with boiling water, digesting the concentrated solution with lead oxide to remove gum, tannic acid, etc., filtering, treating with hydrogen sulphide, and evaporating the clear solution to the consistency of syrup. On cooling after this process, the arbutin crystallizes out in needles, having a bitter taste, and full solubility in water. *Emulcin* or dilute acids decompose it into *glucose*, and *hydroquinone* or *arctuvin:*

$$\underset{\text{Arbutin.}}{\left.\begin{matrix} C_6H_{11}O_5 \\ C_6H_4(OH) \end{matrix}\right\} O} + \underset{\text{Water.}}{H_2O} = \underset{\text{Glucose.}}{C_6H_{12}O_6} + \underset{\text{Arctuvin.}}{C_6H_4(OH)_2},$$

which crystallizes in colorless, fusible and sublimable prisms, soluble in water, alcohol and ether.

Arbutin is converted by concentrated nitric acid into *dinitroarbutin:*

$$\underset{\text{Arbutin.}}{\left.\begin{matrix} C_6H_{11}O_5 \\ C_6H_4(OH) \end{matrix}\right\} O} + \underset{\text{Nitric acrid.}}{(HNO_3)_2} = \underset{\text{Dinitroarbutin.}}{N_2C_{12}H_5(OH)_{13}}$$

crystallizing in pale yellow needles, which, by boiling with dilute sulphuric acid, is resolved into *glucose* and *dinitrohydroquinone:*

Dinitroarbutin + Sulph. acid + Water = Glucose + Water + dinitrohydroquinone.

$$\left.\begin{matrix} C_6H_5 \\ C_6(OH)_{13} \end{matrix}\right\} N_2 + H_2SO_4 + H_2O = C_6H_{12}O_6 + (H_2O)_2 + N_2C_6O_2(OH)_4,$$

a compound of the halloid substitution products of phenol, which crystallizes in golden-yellow plates, dissolving with a blue color in alkalies.

Previous uses: Epigæa has been used in a decoction (the dose being a wineglassful) for strangury, and the relief of vesical catarrh, by the so-called *regular* practitioners, and empirically for the following symptoms:

1. Burning in the neck of the bladder when urinating.
2. Tenesmus of the bladder after urination.
3. Increased flow of pale, limpid urine, with bloody sediment.
4. Urine contains mucus and pus.
5. Discharge of small brown particles resembling fine sand.
6. Dysuria from various causes.

### THE PROVING.

Ch. Mh., male, aged twenty-seven, sanguine temperament, light complexion and hair, blue eyes, tall, muscular and quick motioned, in excellent health in every way.

June 10th, 3 P. M., took 60 ♏ *Φ*. This dose was followed in one hour by loud rumblings in the abdomen, and belchings of flatus at 6 P. M.; took lunch, which was immediately followed by the regurgitation of liquid from the stomach, tasting like thin buttermilk, followed by a feeling in the throat as if more would follow; these risings continued at short intervals, until 8 o'clock; at 10 P. M., sharp flashings of pain along the line of the left ureter, feeling as if small, sharp crystals were rapidly passing downward, for twenty minutes.

At 10.30 P. M. the loud rumblings again occurred in the intestines, with sharp colic pains about the umbilicus, these lasted for about fifteen minutes, and were then relieved entirely by an anal passage of flatus. The following morning, though I arose refreshed as usual at 6 o'clock, I felt a great disinclination to dress myself, and remained with only my shirt and pantaloons on, until my office hours at 8 o'clock; a feeling of shiftlessness in regard to dress only. Constant nausea until breakfast was taken. At 7.30 I again experienced an attack of the cutting pains along the left ureter, lasting fifteen minutes, and followed by a dull pressive pain in a small spot midway between the left iliac crest and the twelfth rib, as if some small blunt instrument were being forced in there; this lasted some twenty minutes, and was followed by soreness about the spot. At 11 o'clock an irresistible desire to sleep came on, and, giving in to it, I lay and slept quietly until noon. On waking refreshed I found that all my teeth felt sore and elongated, especially, though, the molars, and a pain as if bruised was experienced in the occipital and the mastoid processes of the temporal bones; after dinner all pains disappeared until evening, when, after tea, the sour eructations, tasting of buttermilk, again came on, but lasted only a few moments.

June 12th. Again this morning I felt the shiftlessness in regard to dressing myself, and went about the house barefoot until 8.30; nausea until breakfast, after which I again took 60 ♏ Φ, which was followed in one hour by the sharp pains along the left ureter, rumblings of wind in the abdomen, and colic pains, confined to the left iliac region. At 11 A. M., again the desire to sleep came on, and, though not fatigued, I lay down and immediately slept. When I awoke at 12 M. all symptoms had disappeared, and none occurred until after tea, at 6 o'clock, when the sour regurgitations recurred, lasting twenty minutes.

June 13th was a repetition of the previous day, as also was the 14th, though I repeated the dose at 8.30 that morning. The symptoms until 10 P. M. on the 15th, were the same as those of the 13th and 14th, when at this hour I experienced a pain in the left shoulder blade that caused the sweat to break out upon my forehead and hands; it was as if a pair of iron pincers were crushing the superior portion of the scapula with terrible pressure. I could do nothing for half an hour but groan and nurse the part, at the end of which time the pain gradually passed into soreness, which, in turn passed away in about two hours.

At 11.30 a series of sharp knife-like pains developed at the meatus urinarius, which lasted for twenty-five minutes.

During the last five days of the proving my face had the appearance of having been freshly sun-burned, dryness of the mouth, and thirst now constant, as also was an entire loss of all sexual desire.

*Stool.*—Constipation, only one passage during the first forty-eight hours, which was hard, yellow, large and painless. One similar passage at 9 A. M., each day followed. At no time was there a real desire for stool. I sat merely because it seemed necessary that I should have a passage, and at such times only did I gain one.

*Urine.*—The urine during the proving was voided without pain, but only half the frequency of health, and became cloudy or opalescent from the moment of its voidance. The sp. gr. decreased, while the urine became highly acid, and paler than normal.

URINE TABLE.

| HEALTH. | | | | | | | PROVING. | | | | | | |
|---|---|---|---|---|---|---|---|---|---|---|---|---|---|
| Days. | 1 | 2 | 3 | 3 | 5 | 6 | Days. | 1 | 2 | 3 | 4 | 5 | 6 |
| Amount, ℥ | 40 | 32 | 28 | 24 | 28 | 34 | Amount, ℥ | 36 | 24 | 16 | 38 | 38 | 36 |
| Frequency, | 4 | 4 | 4 | 4 | 4 | 4 | Frequency, | 3 | 2 | 1 | 2 | 2 | 2 |
| Color, . . . . . | Deep amber. | | | | | | Color, . . . . . | Pale, opalescent. | | | | | |
| Acidity, . . . | Slight. | | | | | | Acidity, . . . | Highly. | | | | | |
| Appearance, . | Clear. | | | | | | Appearance, . | Cloudy. | | | | | |
| Sp. gr., 10 | 27 | 30 | 28 | 30 | 30 | 33 | Sp. gr., 10 | 30 | 25 | 26 | 24 | 22 | 20 |

No sediment at any time.

